# Supplementary material for: Paradoxical Induction of ALOX15/15B by Cortisol in Human Amnion Fibroblasts: Implications for Inflammatory Responses of the Fetal Membranes at Parturition
Source: Int J Mol Sci. 2023 Jun 29;24(13):10881. doi: 10.3390/ijms241310881 (PMC10341463; doi:10.3390/ijms241310881)
Supplement: Supplementary file 1 [file ijms-24-10881-s001.zip › ijms-2458275-supplementary.pdf]

Contents:

Figure S1 Bioinformatic analysis of the promoter regions of *ALOX15* and *ALOX15B* genes.

Figure S2 Bioinformatic analysis of the promoter region of *EP300* gene.

Table S1 Demographic and clinical characteristics of recruited pregnant women.

Table S2 Primer sequences used in qRT-PCR and ChIP.

Table S3 Antibody information.

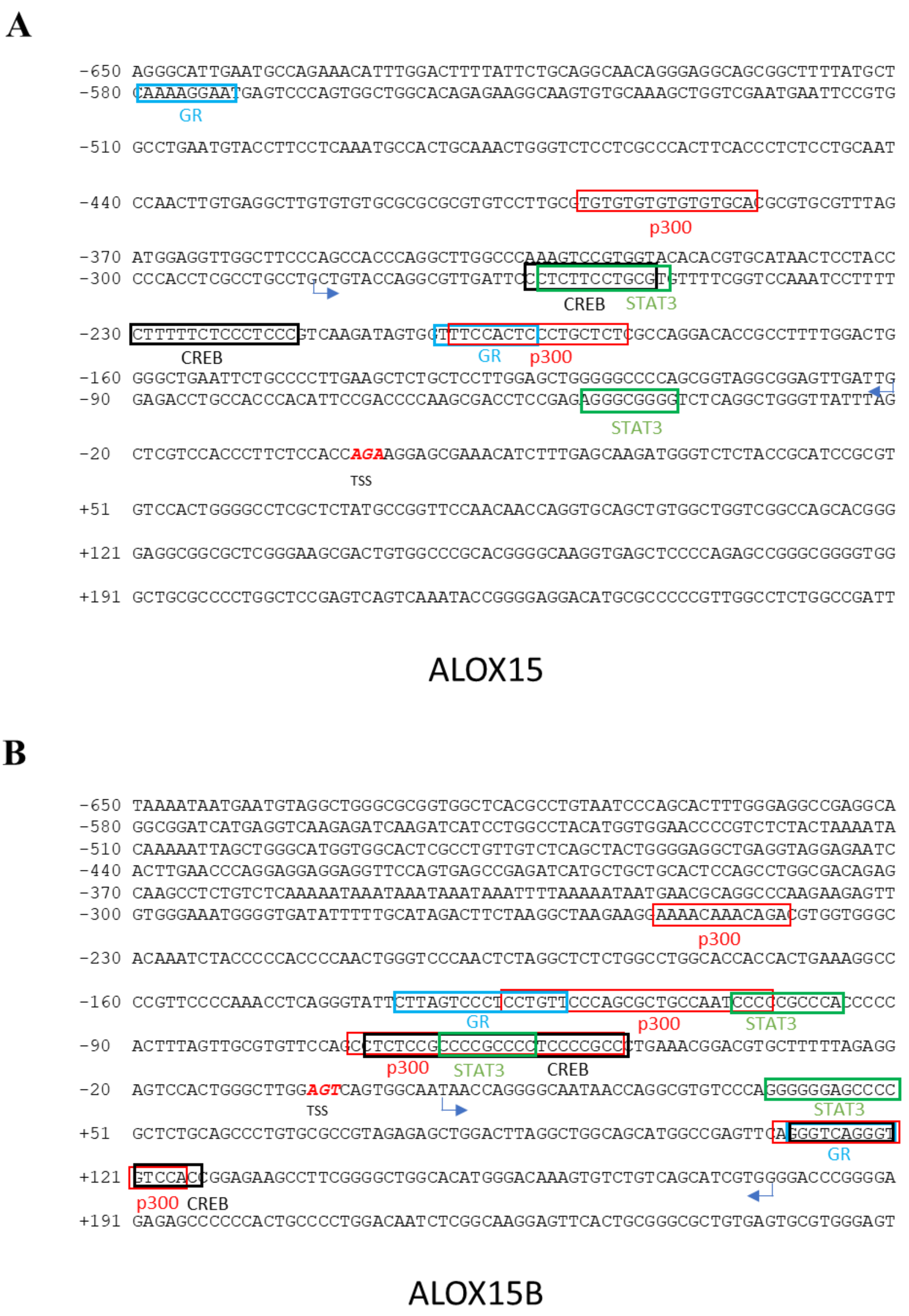

Figure S1. Bioinformatic analysis of the promoter regions of *ALOX15* and *ALOX15B* genes. (A and B) Bioinformatic analysis of the promoter regions (-650bp ~ +250bp) of *ALOX15* (A) and *ALOX15B* (B) genes revealed multiple putative binding sites for GR and p300. Blue box: GR binding sites; Red box: p300 binding sites. Arrows indicate primer aligning positions in ChIP assay. TSS, transcription start site.

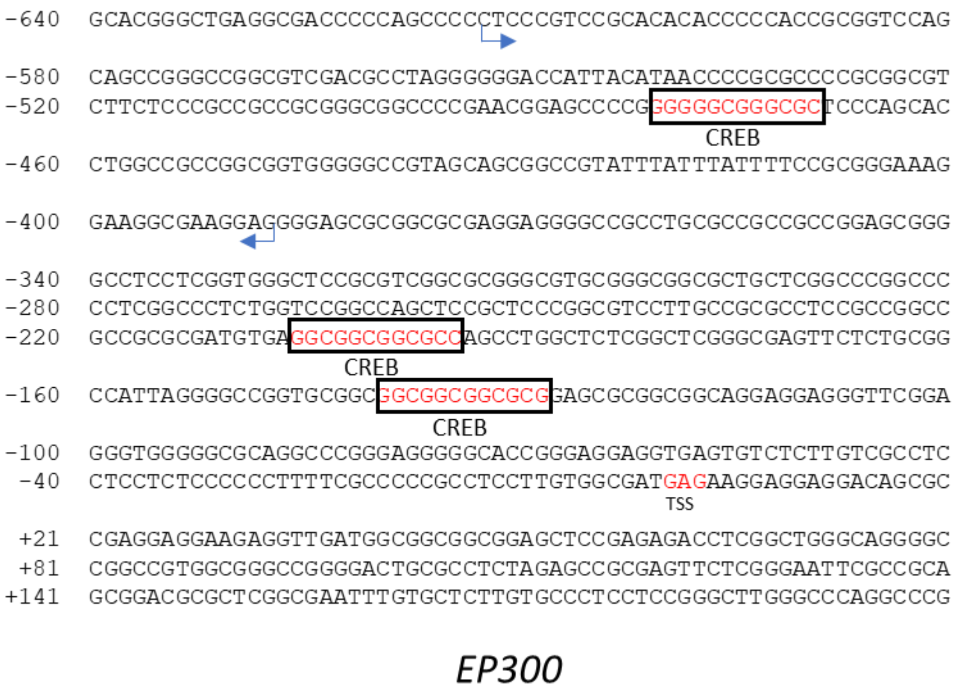

Figure S2. Bioinformatic analysis of the promoter region of *EP300* gene. Bioinformatic analysis of the promoter regions (-640bp ~ +200bp) of *EP300* gene revealed multiple putative binding sites for CREB. Boxed red letters indicate putative CREB binding sites. TSS, transcription start site. Arrows indicate primer aligning positions in ChIP assay.

Table S1. Demographic and clinical characteristics of recruited pregnant women

	TNL (n=7)	TL (n=7)	*P* value
Maternal age (year)	33.00±2.52	32.00±2.58	0.48
Gestational age at delivery (week)	38.96±0.23	39.00±0.49	0.85
Delivery mode			
Vaginal (%)	0	100%	N/A
C-section (%)	100%	0	N/A
Membrane rupture			
SPOM (%)	0	100%	N/A
ARM (%)	100%	0	N/A
Gravidity median (range)	2 (1-5)	2 (1-2)	0.27
Parity median (range)	1 (1-2)	1 (1-2)	0.12
Fetal gender (male/female)	3/4	3/4	0.78
Birth weight (g)	3395.29±207.12	3255.00±143.03	0.17

TNL, term not in labor; TL, term in labor; ARM, artificial rupture of membranes; C-section, caesarean section; SROM, spontaneous rupture of membranes with labor. Maternal age, gestational age at delivery, and birth weight were expressed as mean ± SD and analyzed with unpaired Student's t-test. Gravidity and parity were expressed as median (min–max) and analyzed with the Mann–Whitney U test. Fetal gender was analyzed with Chi-square test. Significance was set at $P < 0.05$.

Table S2. Primer sequences used in qRT-PCR and ChIP.

	Gene	Forward Primer (5'-3')	Reverse primer (5'-3')
qRT-PCR	*ALOX15*	GAAATTAACGTCCGGGCCA	CGATTCCTTCCACATACCGAT
	ALOX15B	CTTCATAGCCACCCTCCCAC	AGGGGCCTTTGGTCTCCA
	EP300	TCCGCCAGCGATGGCACAGA	AGGGGAACTACCAGATCGCAGC
	GAPDH	CCCCTCTGCTGATGCCCCCA	TGACCTTGGCCAGGGGTGCT
ChIP	*ALOX15*	CTGTACCAGGCGTTGATTCC	AATCAACTCCGCCTACCGCT
	ALOX15B	AATAACCAGGCGTGTCCCAG	CCACGATGCTGACAGACACT
	EP300	CTCCCGTCCGCACACAC	CTCCTTCGCCTTCCTTTCCC

Table S3. Antibody information

Antigen	Company	Catalog #	RRID	Dilution Concentration			
				WB	IHC/IF	ChIP	CoIP
**ALOX15**	Thermo Fisher	MA5-25853	AB_2722848	1:500	1:100		
**ALOX15B**	Thermo Fisher	PA5-97456	AB_2812073	1:500	1:100		
**P300**	Abcam	ab14984	AB_301550	1:200			1:50
**t-CREB**	Cell Signaling	9104S	AB_490881	1:1000		1:50	
**p-CREB (Ser^{133})**	Cell Signaling	9198S	AB_2561044	1:1000			
**t-STAT3**	Cell Signaling	9139S	AB_331757	1:1000		1:50	
**p-STAT3 (Tyr^{705})**	Cell Signaling	9145S	AB_2491009	1:1000			
**Lamin A/C**	Cell Signaling	4777S	AB_10545756	1:1000			
**GAPDH**	Proteintech	60004-1	AB_2107436	1:10000			
**H3K27ac**	Abcam	ab4729	AB_2118291			1:100	
**GR**	Cell Signaling	12041S	AB_2631286	1:500		1:50	
